# Science and the War on Truth and Coronavirus

**DOI:** 10.3389/fmed.2020.00563

**Published:** 2020-09-11

**Authors:** Geoffrey P. Dobson

**Affiliations:** Heart, Trauma and Sepsis Research Laboratory, College of Medicine and Dentistry, James Cook University, Townsville, QLD, Australia

**Keywords:** science, coronavirus, truth, university, society, funding

I am a scientist and this is my brief on what the world needs to know about science and COVID-19. Science is a method of truth-telling about the physical world and ways to improve quality of life. It is the most powerful enterprise that has led to improved healthcare, a more sustainable environment, a safer world, and a better “knowing and understanding” about the world we live in. Science is fun and spectacular. And it has rarely let us down, until now.

Despite multiple warnings in 2015, the current global pandemic has revealed major deficits in our preparedness for a viral attack. Governments have let the public down by not supporting early warning programs and for not providing sufficient science funding to understand how different people respond differently to a viral attack, and vaccine development. The present pandemic has also revealed that science underpins a country's national security in ways never appreciated before. The resultant economic upheaval has thrown global supply chains, stock markets, the airline industry, oil markets, and the central bank into frenzied disarray (1). It is regrettable that it took a global pandemic, and the most powerful global economies to come crashing to their knees, with hundreds of thousands of lives lost, to bring science out of the shadows, and into the spotlight.

For decades, politicians have conveyed to the public how their country is leading science innovation and technology *but they fail to sufficiently support it*. In many areas, they are deaf to the calls from scientists, universities and research institutions to increase funding. Scientists themselves must also do a better job at explaining what they do and how science works. What most people don't understand is that science begins with a question and ends with a question (2). This is often confusing. If science is open-ended, how does it solve problems, like those from COVID-19? The short answer is you first have to understand what a virus is, where it has come from, how it enters the body, what it does when it gets there, and finally how to remove it. Finding answers to these questions raises more questions, and *it is this process of knowledge-building* and self-correction, that leads to improved understanding and development of new therapies, vaccines, and technological advances. Unlike bacteria that can thrive almost anywhere, a virus needs a living animal's cellular machinery for its replication and survival. Understanding how a virus has evolved the “tricks” to enter the body “undetected” is not fully understood. And if the COVID-19 virus enters our bodies, why do some people die a horrific death, others have flu-like symptoms, and 20 to 50% become asymptomatic carriers (3, 4)? And why do some children, a few weeks after contracting COVID-19, suffer a hyper-inflammatory attack and succumb to cardiovascular complications and toxic shock? (5).

Before science can answer questions on COVID-19, scientists need to better understand how the immune system works (6). The basic question on why some people have a very mild response, and others die from an explosive inflammatory attack is the 64-billion-dollar question. We have been working on *new drugs to bolster a patient's defense to a pathogen or injury, as part of the stress response, which we believe is controlled by the brain and resides at the intersection of the immune and inflammatory systems* (7, 8). While governments are spending billions of dollars developing a COVID-19 vaccine, we should not get complacent. Notwithstanding the global importance of developing a successful vaccine, it will not answer these, and related, fundamental scientific questions. Nor is a vaccine a substitute for the need to increase science funding, because history will repeat itself, another pandemic will occur, and the cycle will start over again. What we urgently need are new frontline drugs to blunt uncontrolled inflammation, and prevent pulmonary and cardiovascular dysfunction, coagulopathy and metabolic impairment (8–10).

I argue here that the current financial and health crisis is a symptom of a decade or more of budget cuts to basic scientific research, lack of job security among scientists, and declining interest in the next generation to pursue a career in science. Once a research grant is submitted to a funding body, the current success rate in the USA, Australia, UK and Europe is around 5 to 10%, so why facing these odds would a 12th grader or young university student want to become a scientist? The scientist and science, like teachers and nurses, continue to be undervalued by society. In the past decade, funding for the US National Institutes of Health (NIH) has steadily declined, losing around 20% of its funding capacity due to budget cuts, sequestration, and the impact of inflation. President Trump has proposed another 7% drop in NIH funding in 2021, and similar reductions to other science-based agencies (11). In Australia, government investment into research and development is at its lowest in 40 years (12). In Europe, a giant research programme, known as Horizon Europe, will be launched in 2021 across its 28 member states, and other countries, to fund Big Science involving large research groups (13), which leaves the individual scientist or small group of collaborators at a distinct disadvantage. It also remains to be seen, what percentage of those funds will be earmarked to support basic research, and early career scientists to set up their laboratories, who otherwise find it challenging to join large collaborative groups (13). Big Science is not the answer, and history has shown that most discoveries are made serendipitously by individual scientists thinking outside-the-box (2, 14, 15).

Notwithstanding the relentless hyperbole by government officials on funding, and their increasing attempts to pass the torch to industry, many scientists, universities and research institutions are in “survival mode” because of cut-backs. Universities are not businesses in a strict sense; they are involved in teaching and learning, research and technology, and job creation, which are designed to serve the needs of society. Industry, does, however, eventually benefit as the final receiver of potentially translatable products, but they are rarely the primary funders. *Hopefully, the current pandemic will drive home to politicians and lawmakers the societal role of a university, and that the current funding schemes are not fit for purpose*.

Another critical aspect of science is that a “truth” or “fact” in science is an evidence-based statement, not just a “subjective” feeling or an impression (2). When President Donald Trump told the world that he thinks the antimalarial drug hydroxychloroquine is safe and that he would take it, is not an evidence-based statement. An evidence-based statement needs to be tested using the tools of science and medicine, which involves some kind of effect, measurement, human trials and peer review. That CO_2_ in our atmosphere is rising has also sparked a lot of political and public confusion with mixed messages. The *preponderance of evidence* from the vast majority of scientists *specializing in this research* conclude that the rise in CO_2_ is associated with global warming and is accelerated from the burning of fossil fuels, deforestation and human activity (16). Of course, there are critics, however, *the preponderance of evidence* suggests that time is running out, and the warnings that are eerily similar to those leading up to the present pandemic. Unexpectedly, the current global shutdown in early 2020 has also provided us with a global experiment in reducing greenhouse gas emissions. Some countries like China decreased emissions by up to 25%, and the people in India can now see the snow-capped peaks of the Himalayas for the first time in decades (17). It is important to remember that the statement “The rise in CO_2_ is associated with global warming and human activity” is not an absolute statement–it is based on the preponderance of available evidence. Science does not deal with absolutes or first causes, which is its power not its weakness. A provisional-based knowledge allows science to self-correct with improved “truths” and deliverables. This provisional nature of science is often used to attack the process in the media, which sends mixed messages to the public and politicians.

Since February 2020 I have never heard the word “science” mentioned so many times in my 30 years as a scientist, or have I witnessed its credibility being blindly attacked for political gain. We live in a dangerous world and we are outnumbered; 20 million viruses can fit on the head of pin. We need to embrace these new realities, listen to the experts, and not be swayed by the uninformed or naysayers (18–20). Now is a pivotal time in history. I hope the current COVID pandemic has exposed major gaps in Government funding of basic science, and that they stop throwing out pocket-change to scientists thinking that the problem will go away. If we do not learn from our mistakes, I fear 100 years from now historians will write: “the people of the early twenty-first century remained imprisoned by the past and failed to embrace the tools to break free.” Breaking free requires a new global stewardship, with new partnership programs in education, increased funding of basic science and technology, and a renewed optimism that anything is possible.

## Author Contributions

The author confirms being the sole contributor of this work and has approved it for publication.

## Conflict of Interest

The author declares that the research was conducted in the absence of any commercial or financial relationships that could be construed as a potential conflict of interest.
